# Natural hybrid silica/protein superstructure at atomic resolution

**DOI:** 10.1073/pnas.2019140117

**Published:** 2020-11-23

**Authors:** Stefan Görlich, Abisheik John Samuel, Richard Johannes Best, Ronald Seidel, Jean Vacelet, Filip Karol Leonarski, Takashi Tomizaki, Bernd Rellinghaus, Darius Pohl, Igor Zlotnikov

**Affiliations:** ^a^B CUBE - Center for Molecular Bioengineering, Technische Universität Dresden, 01069 Dresden, Germany;; ^b^Institut Méditerranéen de Biodiversité et d’Écologie Marine et Continentale (IMBE), CNRS, Aix-Marseille Université, Université d’Avignon, 13007 Marseille, France;; ^c^Institut de Recherche pour le Développement (IRD), Station Marine d’Endoume, 13007 Marseille, France;; ^d^Swiss Light Source, Paul Scherrer Institute, 5232 Villigen, Switzerland;; ^e^Dresden Center for Nanoanalysis (DCN), Center for Advancing Electronics (cfaed), Technische Universität Dresden, 01069 Dresden, Germany

**Keywords:** biomineralization, sponges, protein crystallography, silica

## Abstract

Formation of highly symmetric skeletal elements in demosponges, called spicules, follows a unique biomineralization mechanism in which polycondensation of an inherently disordered amorphous silica is guided by a highly ordered proteinaceous scaffold, the axial filament. The enzymatically active proteins, silicateins, are assembled into a slender hybrid silica/protein crystalline superstructure that directs the morphogenesis of the spicules. Furthermore, silicateins are known to catalyze the formation of a large variety of other technologically relevant organic and inorganic materials. However, despite the biological and biotechnological importance of this macromolecule, its tertiary structure was never determined. Here we report the atomic structure of silicatein and the entire mineral/organic hybrid assembly with a resolution of 2.4 Å. In this work, the serial X-ray crystallography method was successfully adopted to probe the 2-µm-thick filaments in situ, being embedded inside the skeletal elements. In combination with imaging and chemical analysis using high-resolution transmission electron microscopy, we provide detailed information on the enzymatic activity of silicatein, its crystallization, and the emergence of a functional three-dimensional silica/protein superstructure in vivo. Ultimately, we describe a naturally occurring mineral/protein crystalline assembly at atomic resolution.

## Significance

Using hybrid silica/protein templates, nature has mastered the fabrication of extremely complex macroscopic glass assemblies. Highly symmetric skeletal elements in demosponges are formed following a unique biomineralization mechanism in which polycondensation of an inherently disordered amorphous silica is guided by highly ordered proteinaceous filaments. Here we provide a comprehensive three-dimensional atomistic view of this hybrid assembly. The structure, occurring in the crystalline form in vivo, was measured in situ using the serial crystallography method. Together with a high-resolution transmission electron microscopy and energy-dispersive X-ray spectroscopy study, we provide structural, chemical, and functional information on a naturally forming hybrid mineral/organic crystal.

**Fig. 1.**
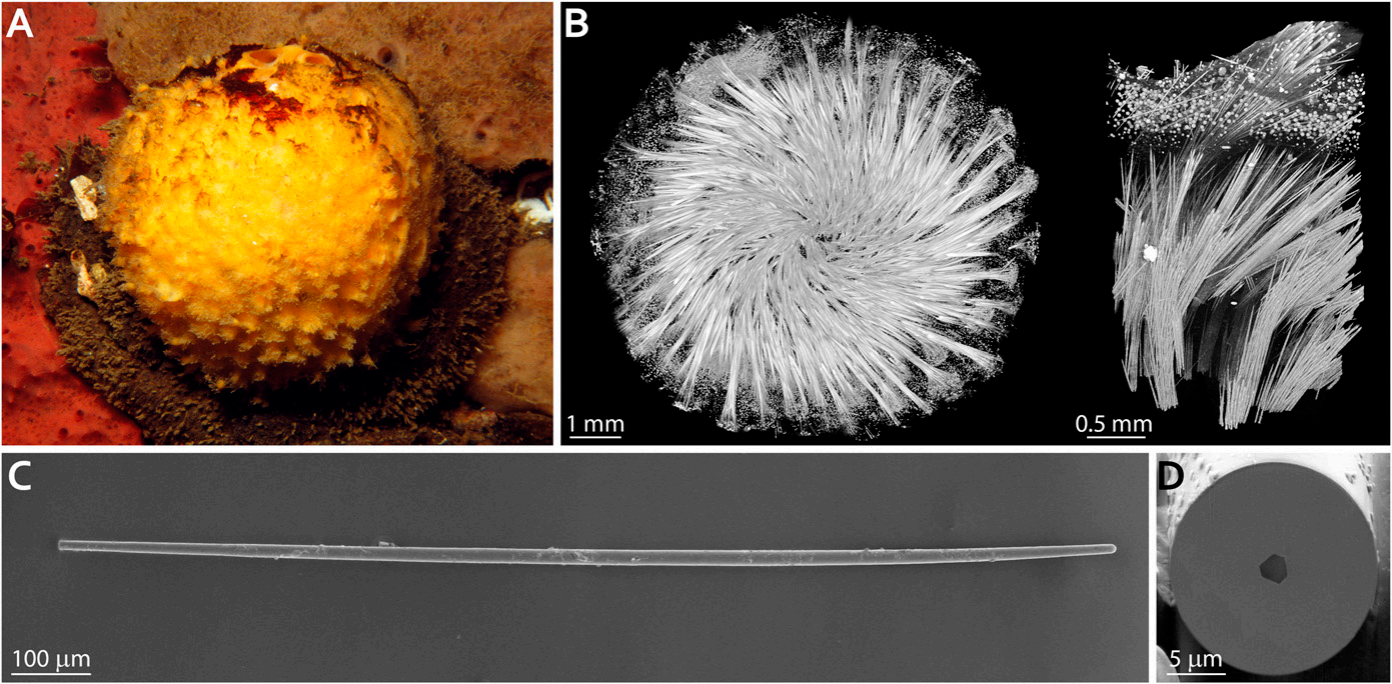
Skeletal elements of the demosponge *T. aurantium*. (*A*) The demosponge *T. aurantium*. (*B*) 3D visualization of the mineralized silica skeleton in *T. aurantium* obtained using X-ray microtomography. (*Left*) An entire sponge skeleton. (*Right*) A crop out of the cortical part of the sponge. (*C*) A single needle-like spicule (strongyloxea) extracted from the cortical region of *T. aurantium*. (*D*) A cross-section through the spicule in *C*, cut using FIB milling, demonstrating the presence of an axial filament going through the center of the spicule.

Demospongiae is a diverse class of sponges (phylum Porifera) consisting of more than 7,400 species (1). Dating back to the Cryogenian period, these animals are among the first multicellular organisms to inhabit the Earth (2). Demosponges have a significant role in reef ecology and reef bio-erosion and are a leading metazoan class for the discovery of novel bioactive chemicals (3, 4). However, what is arguably most remarkable about these organisms is the fascinating diversity of highly regular morphologies of individual skeletal elements, called spicules, that comprise the humble body plan of the various species and their unique mineralization mechanism (5, 6). Skeletogenesis of these sessile animals—spicule formation, transport and fixation in the tissue—is orchestrated by a number of different cells through a series of cellular and molecular mechanisms (7). In demosponges, the spicules are made of hydrated amorphous silica and ranging in size from a few microns to centimeters (8). Astonishingly, being composed of an inherently disordered amorphous material, these skeletal elements exhibit a variety of highly regular species-specific branched morphologies and are considered a paradigm of symmetry in biomineralized systems (9). A recent study revealed that this order is realized with the assistance of axial filaments that pass through the center of every branch in every spicule and direct silica deposition (10). The filaments in demosponges were demonstrated to be perfect slender protein crystals, and their branching on well-defined crystallographic planes of that crystal was shown to be responsible for the high spatial regularity and symmetry of the spicules. The periodic structure in the axial filaments in demosponges was first noted in 1969 by Garrone (11), long before the identification of the protein family that comprise them (12). In 1998, using the common marine demosponge *Tethya aurantium* as a model system (Fig. 1*A*), Morse and coworkers (12) reported the discovery of silicateins, the protein family associated with the filaments.

Silicatein is the first discovered enzymatically active multifunctional protein that catalyzes the polymerization of biogenic silica and is key to its subsequent condensation and shaping (13–15). Initially, three isoforms—silicatein-α, silicatein-β, and silicatein- γ, in a molar ratio of 12:6:1 and molecular masses of 29, 28, and 27 kDa, respectively—were observed in the axial filaments of *T. aurantium* (12). Using a close homology to the cathepsin protein family—sequence identity between silicatein-α in *T. aurantium* and metazoan cathepsin L is 52%—silicatein’s mechanism of catalysis was modeled. It is suggested that, analogous to the hydrolysis of peptide bonds in the cysteine proteases by cathepsin, a catalytic triad composed of Ser, His, and Asn (a serine protease) is key to the polycondensation of silicic acid by silicatein-α (13, 16). In fact, using native and recombinant silicatein, this mechanism was shown to be efficient in the synthesis of a wide range of technologically important materials in ambient conditions beyond the silica mineral. These include a number of silicones, a large number of oxides and carbonates (e.g., TiO_2_, γ-Ga_2_O_3_, ZrO_2_, CaTiO_3_, CaCO_3_), perovskites (BaTiOF_4_), and more (17–22). Nevertheless, despite its biological and biotechnological importance, due to difficulties in producing and crystallizing recombinant silicatein in vitro, a detailed high-resolution structure of silicatein, and thus a refined mechanism of silica polycondensation, has remained unknown (23).

In contrast, the crystalline assembly of silicatein units in vivo, inside the axial filaments of demosponges, such as *T. aurantium*, is well recorded by a number of recent X-ray diffraction and transmission electron microscopy studies (10, 24–26). The marine sponge *T. aurantium* (Fig. 1*A*) is a common model system for studying biomineralization in siliceous sponges. The body of this sponge contains two main types of skeletal glass elements: bundles of needle-shaped spicules (strongyloxeas) radiating from the center of the sponge and star-shaped spicules (asters) occupying the cortical part of the animal (Fig. 1*B*). The former compose almost 75% of the dry weight of the entire organism, and individual spicules can be >2 mm in length and >30 µm in diameter (Fig. 1*C*) (12). A cross-section through a strongyloxea demonstrates the threefold symmetry of an ∼2-µm-thick axial filament at the center of the spicule (Fig. 1*D*). Silicateins in the filaments of these glass needles were shown to be packed in a perfect hexagonal superstructure with lattice parameters of *a* = 5.95 ± 0.01 nm and *c* = 11.89 ± 0.01 nm, with the *c*-axis oriented parallel to the long axis of the spicule (10). Yet again, whereas a number of contradictory models describing the mechanism of silicatein self-assembly were previously proposed, the lack of its tertiary structure limits our understanding of silicatein crystallization, its assembly into a filament, spicule morphogenesis, and, finally, demosponge skeletogenesis (27, 28).

**Fig. 2.**
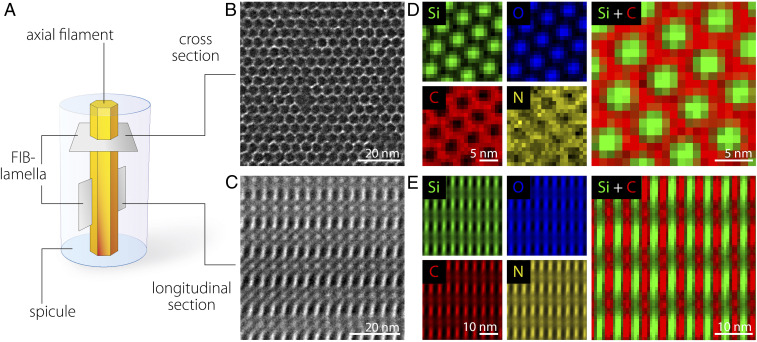
HRTEM analysis of the axial filament. (*A*) Schematic representation of the studied spicule and the samples prepared by the FIB milling method. (*B* and *C*) HRTEM images obtained by studying the cross-section (along the [001] zone axis) (*B*) and the longitudinal section (along the [100] zone axis) (*C*) of the axial filament as depicted in *A*. (*D* and *E*) High-resolution EDX maps obtained by measuring the cross-section and the longitudinal section of the axial filament as depicted in *A*, respectively. The composite images on the right summarize the signals from silicon (Si) and carbon (C) atoms only.

Detailed structural and compositional analysis of the axial filament in the needle-like spicule from *T. aurantium* was performed using high-resolution transmission electron microscopy (HRTEM) and energy-dispersive X-ray spectroscopy (EDX), respectively (Fig. 2). The crystalline nature of silicatein in the filament was studied by preparing two types of lamellas using the focused ion beam (FIB) milling method, one cut perpendicular and the other cut parallel to the long axis of the filament: cross-sectional and longitudinal cuts, respectively (Fig. 2*A*). Fig. 2 *B* and *C* show the extraordinarily ordered and at the same time complex substructure of the filament. In cross-section (Fig. 2*B*), as expected, a highly periodic hexagonal pattern consisting of a dark phase meshed by a bright honeycomb structure is observed; however, when looking at the crystal using the longitudinal cut (Fig. 2*C*), a nontrivial intricate configuration emerges. Chemical analysis in both directions indicates that the axial filament is in fact a hybrid structure composed of an organic matter, presumably silicatein, and silicon oxide. In Fig. 2 *D* and *C*, EDX analysis in the two directions differentiates between two unique zones: one rich in silicon (green) and oxygen (blue) atoms and the other rich in carbon (red) and nitrogen (yellow) atoms.

When comparing the composite EDX images with TEM data, it is evident that in cross-section (Fig. 2 *B* and *D*), the observed honeycomb-like mesh (bright zones in Fig. 2*B*) is mostly organic and is filled with silica (dark inclusions in Fig. 2*B*). Similarly, in the longitudinal cut (Fig. 2 *C* and *E*), the very bright areas in TEM are rich in the organic proteinaceous material, and the very dark areas are filled with the silicon oxide. However, additional gray domains suggest an intricate superposition of the two main components in these areas. Whereas similar intricate TEM images were obtained in previous studies of axial filaments in demosponges (24), the three-dimensional (3D) architecture of this hybrid crystalline structure was never elucidated.

**Fig. 3.**
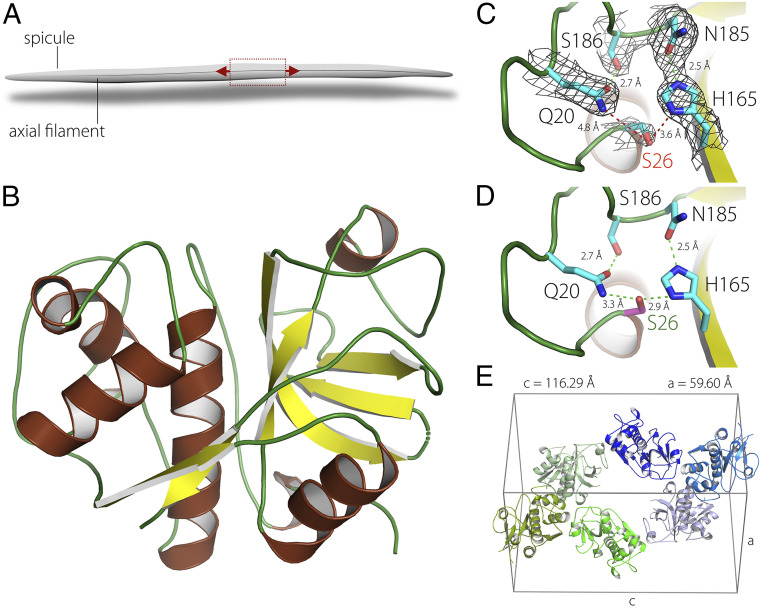
Protein crystallography study of silicatein. (*A*) Schematic representation of a single spicule and the rastering direction during the serial crystallography experiment. (*B*) Tertiary structure of silicatein-α. (*C*) The catalytic triad in silicatein in its conformation as measured by the protein crystallography experiment. (*D*) Hypothesized active conformation of the catalytic triad in *C*. (*E*) A single unit cell of silicatein crystal belonging to the symmetry group *P3*_*1*_*21* with *a* = 5.96 nm and *c* = 11.63 nm.

Nevertheless, being surrounded by a thick layer of an amorphous material, the skeletal elements as formed by the living organism are ideal samples for use in a high-resolution in situ protein X-ray crystallography study of the axial filament. A typical crystallographic study involves the collection of a series of diffraction patterns measured while stepwise rotating a protein crystal (of at least 100 µm) and irradiating it with an X-ray beam. Knowing the angles in which the different diffraction images were obtained allows for reconstruction of the tertiary structure of the protein by analyzing the intensity distribution of the scattered X-rays in all the patterns (29). Initially, this classical approach was used in an attempt to collect a sufficient dataset from the axial filament from strongyloxeas in *T. aurantium* at macromolecular crystallography beamline X06SA at the Swiss Light Source, Paul Scherrer Institute, Villigen, Switzerland. Here the spicules were mounted on a sample holder with their long axis perpendicular to the X-ray beam and parallel to the rotation axis of the holder. Although the measurements were performed in cryogenic conditions, collecting a full dataset was not possible, as the 2-µm-thick axial filament containing the protein crystal was damaged almost immediately after the start of data acquisition. Therefore, an alternative approach based on serial X-ray crystallography was adopted (30). In this method, commonly used to determine the structure of radiation-sensitive protein nanocrystals in X-ray free-electron laser facilities (31), diffraction patterns are collected from numerous randomly oriented crystallites. With a sufficient number of these snapshots, the structure of the protein can be determined using a series of numerical techniques (32). In this work, a total of 3,608 individual diffraction snapshots were collected in cryogenic conditions from 90 different needle-like spicules extracted from the sponge *T. aurantium*. In each spicule, a rectangular beam of 30 µm × 10 µm was rastered along the axial filament to avoid measuring a previously damaged area (Fig. 3*A*). These data were sufficient to generate a complete dataset with a resolution of 2.4 Å (*SI Appendix*, Table S1).

The tertiary structure of silicatein (Protein Data Bank [PDB] ID code 6ZQ3; *SI Appendix*, *Crystallographic Information Files S1 and S2*, containing atom positions and structure factors, respectively), was determined by molecular replacement using the cathsilicatein chimera structure as a search model (PDB ID code 2VHS), which has the highest sequence identity to silicatein-α available in the PDB (33). Using the known sequence of the most abundant isoform of silicatein, silicatein-α (*SI Appendix*, Fig. S1), a well-defined electron density was obtained allowing the reconstruction of a continuous protein model from Pro3 to the C-terminal residue Leu218 (except of loop Ser175 to Gln178) (*SI Appendix*, Table S1). Indeed, the tertiary structure of silicatein resembles the fold of Cathepsin L (PDB ID code 1MHW) (34), with an rmsd of 0.67 Å across 199 Cα positions (Fig. 3*B*). Surprisingly, these data reveal that the postulated active site of the protein—the catalytic triad of Ser26, His165, and Asn185—is in an inactive mode (Fig. 3*C*). The distance between the hydroxyl group of Ser26 and the imidazole ring of His165 is 3.6 Å, too long to allow catalytic conformation (35). It can be hypothesized that a rotation of the Ser26 side chain into another favorable conformation (a distance of 2.9 Å to the imidazole ring of His165) leads to activation of the enzymatic activity of silicatein, which is also supported by an additional hydrogen bond network including Gln20 and Ser186 (Fig. 3*D*) (33). However, in its crystalline form, in the axial filament inside the spicule, the enzymatic activity of the protein is switched off.

**Fig. 4.**
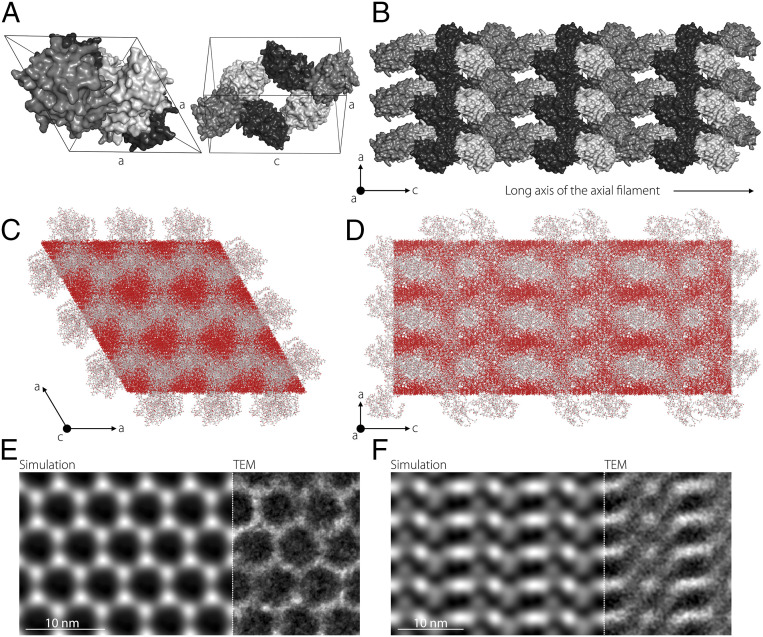
The hybrid silica/silicatein superstructure in the axial filament of *T. aurantium*. (*A* and *B*) Single unit cell (*A*) and the entire assembly (*B*) of the proteinaceous crystal. The individual protein units are shown in gray scale. (*C* and *D*) 2D projections of the atomic structure of the axial filament obtained when looking parallel to the *c*- and *a*-axes of the hybrid hexagonal superstructure, along the [001] and [100] zone axes, respectively. Silicon (Si) and oxygen (O) atoms are color-coded in red; carbon (C), nitrogen (N), and sulfur (S) atoms are color-coded in gray. (*E* and *F*) 2D HRTEM images along the [001] (*E*) and [100] (*F*) zone axes. (*Left*) HRTEM contrast simulations based on the models presented in *C* and *D*. (*Right*) Experimental HRTEM images.

To form the axial filament, the silicatein molecules are packed into a single slender crystal with six proteins in a single hexagonal unit cell belonging to the symmetry group *P3*_*1*_*21* with *a* = 5.96 nm and *c* = 11.63 nm (Fig. 3*E*). A 3D representation of a single unit cell and of the entire axial filament is shown in Fig. 4 *A* and *B*, respectively. However, judging by the homogeneous silica signal obtained in the EDX analysis (Fig. 2 *D* and *E*) and by previous studies demonstrating the presence of amorphous silica inside the filament (24, 25, 36), it is reasonable to postulate that in fact, silica molecules are randomly dispersed to occupy the entire space between the protein units. Based on this assumption, an atomistic model of the hybrid silicatein/silica crystal was built (*SI Appendix*, *Crystallographic Information File S3*). Two-dimensional (2D) projections of the reconstructed crystal when looking parallel to the *c-* and the *a-*axes of the lattice are presented in Fig. 4 *C* and *D*. These data were used to simulate TEM projections of the hybrid structure in both directions. Remarkably, the modeled images obtained parallel to the *c-*axis and the *a-*axis (Fig. 4 *E* and *F*) fully agree with the experimental TEM measurements (Fig. 2 *B* and *C*). This result indicates that the axial filament in demosponges is a naturally occurring dense composite assembly consisting of a silicatein crystal surrounded by a complementary mesoporous silica phase (37). Thus, it provides a cohesive description of the filament and indisputably resolves the ongoing debate over the structure and composition of this superstructure and its role in demosponge spiculogenesis (10, 24–28, 37).

Our data yield a comprehensive 3D view of a hybrid silica/protein crystal that comprises the axial filaments of siliceous skeletal elements in the demosponge *T. aurantium*. The tertiary structure of silicatein-α, occurring in a crystalline form in vivo, was measured in situ by serial crystallography and realized at a protein crystallography beamline at a synchrotron. It provides a detailed description of the enzymatic activity of the protein and sheds light on its assembly into a highly ordered superstructure that guides the morphogenesis of spicules made of intrinsically disordered amorphous silica. Specifically, this information provides the opportunity to better understand the processes that drive filament formation, to identify the driving forces that orchestrate its branching, and, ultimately, to unravel mechanisms that morph amorphous glass into highly symmetric 3D structures at ambient conditions. With this high-resolution TEM and EDX spectroscopy study, we provide structural, chemical, and functional information on a naturally forming hybrid mineral/organic crystal.

## Materials and Methods

### Materials.

For the protein crystallography experiment and HRTEM imaging and analysis, the sponge *T. aurantium* (Pallas, 1766) was collected on May 1991, at 15 m depth in the western Mediterranean Sea near Marseille at the entrance to a cave on Jarre Island. The specimen was fixed in 10% neutral formalin for 2 d and then preserved in 90% ethanol. For the computed tomography (CT) scan, a whole *T. aurantium* specimen was loaned from the Naturkunde Museum Berlin, Germany (wet collection, catalog no. ZMBPor108554), where it was stored in ∼75% ethanol.

### X-Ray Micro-CT.

During CT acquisition, the whole specimen was temporarily fixed in a custom-built container, filled at the bottom with 75% ethanol. Tomographic data were obtained using the EasyTom Nano 160 (RX Solutions) at the Department of Biomaterials, Max Planck Institute of Colloids and Interfaces. The tomographic data reconstruction was done with RX Solutions software. Whole specimen scans were performed at 50 kV source voltage and 200 µA source current, with 1,440 profile images resulting in minimum isometric voxel sizes of 8 µm. A region of interest at the periphery was scanned at higher resolution at 60 kV source voltage and 89 µA source current, with 1,440 profile images resulting in minimum isometric voxel sizes of 1.6 µm. The segmentation and imaging of the CT data were done in Amira-Avizo (Thermo Fisher Scientific).

### Focused Ion Beam Milling.

Lamellas for TEM investigation were prepared with the FIB technique using an FEI Helios 660 dual-beam scanning electron microscope with FIB. To remove residual amorphous and damaged material, low-voltage (900 V) Ar milling was done using the Fischione NanoMill 1040 system.

### TEM.

TEM was conducted using a JEOL JEM F200 transmission electron microscope operated at 200 kV acceleration voltage. Local EDS analysis was performed using a dual 100-mm^2^ windowless silicon drift detector. The EDX spectra were denoised with principal component analyses using three components (38). The signal-to-noise ratio was further improved by applying template-matching algorithms to large-scale elemental images using SmartAlign software (39, 40).

### Serial Protein Crystallography.

X-ray diffraction images were collected at the macromolecular crystallography beamline X06SA at the Swiss Light Source, Paul Scherrer Institute on an Eiger 16M detector (Dectris). An X-ray beam of 1-Å wavelength was focused to 30 µm × 10 µm at the spicules that were cryogenically protected in a 50% ethylene glycol solution. The flux and dose rates were 9.92 × 10^11^ photons/s and 0.021 to 0.033 MGy at 0.1 s exposure time, respectively. Two data collection modes in cryogenic conditions were used. A small portion of crystals were measured with classical rotation method with wedges of 30° to 45°. Although the resolution was poor (d >3.5 Å), these helped confirm the proper space group but did not provide a complete dataset. The majority of images were collected as still images using the fast 2D raster procedure available at the beamline (41). A total of 17,981 images were collected from 90 spicules. All images were processed using the CrystFEL 0.8.0 package (42, 43).

Through a combination of spot finding with the peakfinder8 algorithm and indexing with the XDS, MOSFLM, and XGANDALF algorithms, 3,608 images were labeled as hits and integrated. Indexing ambiguity, present in a *P3*_*1*_*21* space group, was resolved with the Ambigator tool. Afterward, intensities were scaled with a single iteration of the Partialator program, using the Xsphere algorithm to take into account partiality of observed reflections. Scaled reflection intensities were converted to amplitudes and saved to an MTZ file for further processing.

### Protein Structure Determination.

Molecular replacement search model (PDB ID code 2VHS) was adjusted using the PyMOL Molecular Graphics System version 1.73, resulting in one protein molecule without ligands in the asymmetric unit. Initial phases were successfully obtained in the *P3*_*1*_*21* space group by molecular replacement using the MolRep program of the CCP4 suite (44). The initial model was iteratively refined using REFMAC5 (45) and Coot version 0.8.9.2 (46). The statistics for the final model are presented in *SI Appendix*, Table S1. Images of the structure were created using Pymol version 1.73.

### HRTEM Simulation.

All atoms of the protein model with occupancy 0.0 were removed using Coot version 0.8.9.2 (46). The six protein molecules of the asymmetric unit were combined using the Pymol software package, and the symmetry was adjusted to space group *P1*. The asymmetric unit was filled with amorphous silica molecules with 4 Å distance to the protein surface using the MDAnalysis software package (47, 48) and then converted into a CIF format for further analysis (*SI Appendix*, *Crystallographic Information File S3*). Multislice HRTEM contrast simulations were performed using the Tempas 3.0.16 software package. As microscope parameters, a spherical aberration coefficient of 1.0 mm, a spread of defocus of 2 nm, and a convergence angle of 0.15 mrad were used as imaging parameters. A thickness of 70 nm and a defocus of −2,300 nm were chosen to provide the best fit the experimental imaging conditions. To further match the experimental images, a bandpass filter from 0 to 0.0035 Nyquist was applied with an exponential decay.

## Supplementary Material

Supplementary File

Supplementary File

Supplementary File

Supplementary File

Supplementary File

## Data Availability

All study data are included in the main text and *SI Appendix*.
