# Supplementary material for: Natural hybrid silica/protein superstructure at atomic resolution
Source: Proc Natl Acad Sci U S A. 2020 Nov 23;117(49):31088–93. doi: 10.1073/pnas.2019140117 (PMC7733841; doi:10.1073/pnas.2019140117)
Supplement: Supplementary File [file pnas.2019140117.sapp.pdf]

Supplementary Information for

Natural hybrid silica/protein superstructure at atomic resolution

Stefan Görlich, Abisheik John Samuel, Richard Johannes Best, Ronald Seidel, Jean Vacelet, Filip Karol Leonarski, Takashi Tomizaki, Bernd Rellinghaus, Darius Pohl and Igor Zlotnikov

Corresponding Author: Igor Zlotnikov
E-mail: igor.zlotnikov@tu-dresden.de

This PDF file includes:

Figure S1
Tables S1

Other supplementary materials for this manuscript include the following:

Crystallographic Information Files S1-S3

www.pnas.org/cgi/doi/10.1073/pnas.2019140117

Silicatein Alpha Primary Sequence

```
  1 MYLGTLVVLC VLGAAIGEPM PQYEFKEEWQ LWKKQHDKSY STNLEELEKH
 51 LVWLSNKKYI ELHNANADTF GFTLAMNHLG DMTDHEYKER YLTYTNSKSG
101 NYTKVFKREP WMAYPETVDW RTKGAVTGIK SQGDCGASYA FSAMGALEGI
151 NALATGKLTY LSEQNIIDCS VPYGNHGCKG GNMYVAFLYV VANEGVDDGG
201 SYPFRGKQSS CTYQEQYRGA SMSGSVQINS GSESDLEAAV ANVGPVAVAI
251 DGESNAFRFY YSGVYDSSRC SSSSLNHAMV ITGYGISNNQ EYWLAKNSWG
301 ENWGELGYVK MARNKYNQCG IASDASYPTL
```

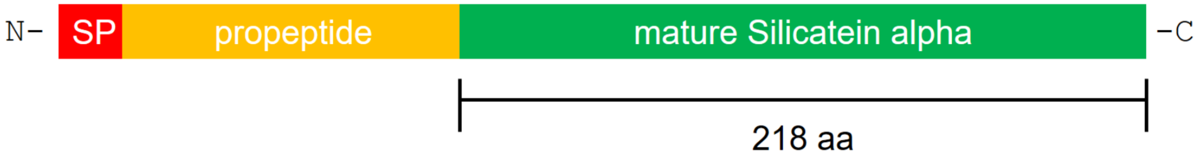

Fig. S1. The protein sequence of silicatein-alpha is present in the Uniprot database under O76238.

Table S1. Data collection and refinement statistics (molecular replacement)

	Silicatein-alpha
**Data collection**	PDB 6ZQ3
Space group	P 3_1 2 1
Cell dimensions	
a, *b*, *c* (Å)	59.60, 59.60, 116.29
α, β, γ (°)	90.0, 90.0, 120.0
Resolution (Å)	51.5 – 2.4 (2.486 – 2.4)*
R_{split} (%)	18 (115)
$I / \sigma I$	6.1 (0.96)
Completeness (%)	100 (100.00)
Redundancy	159 (113)
**Refinement**	
Resolution (Å)	47.18 - 2.40
No. reflections	9827 (973)
R_{work} / R_{free}	0.2381 / 0.2926
No. atoms	1612
Protein	1586
Ligand/ion	0
Water	26
B-factors	52.35
Protein	52.26
Ligand/ion	-
Water	58.12
R.m.s. deviations	
Bond lengths (Å)	0.028
Bond angles (°)	2.12

*90 crystals analyzed. Values in parentheses are for highest-resolution shell.

Data Files:

Crystallographic Information File S1: Tertiary structure of Silicatein - atom positions
Crystallographic Information File S2: Tertiary structure of Silicatein – structure factors.
Crystallographic Information File S3: Atomistic model of the hybrid silicatein/silica structure.
